# Retrospective Analysis of Regorafenib Efficiency in Treatment of Metastatic Colorectal Cancer—Experience of Two Polish Comprehensive Cancer Centers

**DOI:** 10.3390/jcm15010332

**Published:** 2026-01-01

**Authors:** Magdalena Grabiec, Dominika Raźniewska, Tadeusz Kałużewski, Magdalena Krakowska, Barbara Radecka, Piotr Potemski

**Affiliations:** 1 Department of Clinical Oncology, Medical University of Lodz, 92-231 Lodz, Poland; 2 Department of Oncology, Institute of Medical Sciences, University of Opole, 45-040 Opole, Poland; 3 Department of Clinical Oncology, Tadeusz Koszarowski Cancer Center in Opole, 45-061 Opole, Poland; 4 Department of Genetics, Polish Mother’s Memorial Hospital Research Institute, 93-338 Lodz, Poland

**Keywords:** metastatic colorectal cancer, regorafenib, retrospective study, real-word data, survival

## Abstract

**Objectives:** Colorectal cancer is a major public health concern, ranking third in incidence among all malignant tumors both in Poland and globally. We conducted a retrospective study to evaluate the effectiveness of regorafenib in patients with metastatic colorectal cancer ineligible for local therapy treated at two Polish comprehensive cancer centers between 2021 and 2024. **Methods:** The analysis included 29 patients who had previously received all standard therapies: fluoropyrimidines, oxaliplatin, and irinotecan (in a multi-agent regimen or sequentially) and bevacizumab (anti-VEGF therapy). In patients with tumors negative for *KRAS*, *NRAS* and *BRAF* mutations, cetuximab or panitumumab (anti-EGFR therapy) were also used. **Results:** The median progression-free survival (PFS) was 2.5 months, and the median overall survival (OS) was 5.8 months. Disease stabilization was observed in five patients, with a median duration of 5.6 months, and no partial or complete remission were recorded. **Conclusions:** Our results were similar to those of the phase III CORRECT trial, which established the clinical utility of regorafenib. Only minor differences in survival outcomes were noted—likely due to real-world variability in patient characteristics and timing of treatment assessments. However, continued investigation of personalized and sequential treatment strategies that contain anti-angiogenic drugs is warranted to optimize outcomes.

## 1. Introduction

Colorectal cancer represents a significant public health problem, ranking third in terms of incidence among malignant neoplasms both in Poland and globally [1,2]. In Poland, 17,700 individuals were diagnosed with colorectal cancer in 2021 [2].

According to ESMO guidelines, 15–20% of patients present with metastatic disease at the time of diagnosis, and approximately 20–50% of patients with non-advanced disease will develop metastatic disease during the course of their illness [3].

In advanced disease, standard management is based on multi-drug chemotherapy, either combined with targeted agents or administered without them, as well as immunotherapy [3]. At the time of treatment qualification, therapeutic decisions are made considering the patient’s condition, comorbidities, preferences regarding the mode of treatment administration, and consent to potential adverse effects. Another critical factor influencing treatment strategy is the outcome of molecular testing, which includes the identification of mutations in the *KRAS*, *NRAS*, and *BRAF* genes, as well as the assessment of microsatellite instability/mismatch repair status.

Recent advancements in personalized medicine have enabled tailoring therapies to the molecular aberrations of tumors [4]. For patients with the *BRAF* V600E mutation, which occurs in 8–12% of cases, combination therapy with encorafenib and cetuximab can be considered based on the results of the BEACON study [5]. Another molecular aberration for which targeted therapy may be considered is HER2 overexpression, observed in 3–5% of patients [6]. Additionally, the *KRAS* G12C mutation, present in 3% of colorectal cancer cases, allows for the use of dual-drug regimens such as sotorasib and panitumumab, based on the phase III CodeBreak 300 trial [7], or adagrasib and cetuximab, based on the phase I/II KRYSTAL-1 trial [8]. Entrectinib and larotrectinib can be utilized in patients with *NTRK1*, *NTRK2*, or *NTRK3* gene fusions, which occur in less than 1% of colorectal cancer cases [9]. Furthermore, based on the phase I/II basket trial Libretto-001, selpercatinib may be considered for patients with *RET* gene fusions, observed in fewer than 1% of cases [10].

For patients without the aforementioned molecular aberrations who had received all standard drugs and are in very good or good performance status and express a willingness to continue treatment, there are no clear guidelines regarding the continuation of therapy. One of the options to consider is regorafenib, a multikinase inhibitor, targeting kinases involved in tumor angiogenesis and oncogenesis as well as kinases associated with the tumor microenvironment. Regorafenib particularly inhibits mutated KIT kinase, a key driver of oncogenesis, thereby blocking tumor cell proliferation. Its efficacy after all approved standard therapies was confirmed in the phase III CORRECT trial [11]. This study was conducted between 2010 and 2011 across 114 centers in 16 countries. A total of 760 patients were enrolled and randomized in a 2:1 ratio into two groups: the first group received regorafenib, while the second group received a placebo. Both groups were provided with best supportive care. Eligible participants had to have life expectancy exceeding three months. The trial achieved statistical significance for both its primary and secondary endpoints, which were overall survival (OS) and progression-free survival (PFS), respectively.

The aim of our study was to evaluate the efficacy of regorafenib in patients from two Polish oncology centers by assessing PFS and OS, and compare them with the results of the CORRECT trial.

## 2. Materials and Methods

The study included 30 patients with inoperable metastatic colorectal cancer treated with regorafenib between 2021 and 2024 from two oncology centers (The Multidisciplinary Center of Oncology and Traumatology in Lodz and Tadeusz Koszarowski Cancer Center in Opole). One patient was excluded from the analysis as he did not attend the scheduled visit after the first treatment cycle and missed subsequent cycles. His survival exceeded the planned imaging evaluation after 3 months, leading to exclusion. The final analysis was conducted on data from 29 patients. All patients were initially qualified to receive the standard dose of 160 mg once daily for 3 weeks. The drug was reimbursed based on the individual approvals (the Emergency Access to Drug Technology).

Statistical analyses were performed to evaluate PFS and OS within a time-to-event framework. PFS was defined as the time from the start of treatment until disease progression or death from any cause, whichever occurred first. OS was defined as the time from the start of treatment until death from any cause. Patients who were alive at the last follow-up were censored at that time. The analysis included the calculation of median values of PFS and OS, along with the comparison of survival curves using Kaplan–Meier estimation. Data visualization was generated using Python (version 3) with the Matplotlib v3.10 and Lifelines v0.30 libraries.

## 3. Results

The median age of patients included in the study was 64 years. Baseline characteristics are presented in Table 1. Surgical resection of the primary tumor was performed in 72.4% of patients. All patients had metastatic disease, with 69% presenting distant metastases at initial diagnosis and 31% owing to relapse after previous radical local treatment. Prior to regorafenib treatment, 34.5% of patients had received more than three lines of therapy. Molecular testing for *KRAS*, *NRAS*, and *BRAF* mutations, as well as microsatellite instability (MSI), were not performed in one patient due to the difficulties in obtaining reliable material. Additionally, 44.8% of patients underwent HER2 expression testing by immunohistochemistry, all with negative results. None of the patients received immunotherapy due to the absence of microsatellite instability.

**Figure 1:**
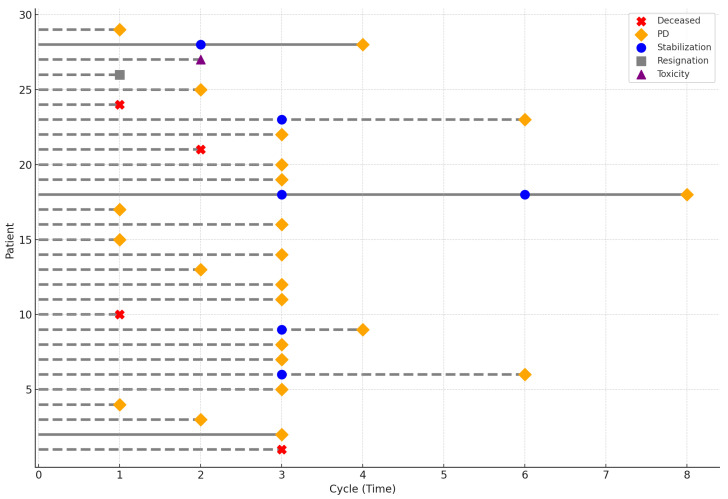
Characteristics of the treatment course. The symbols indicate the reason for treatment discontinuation for each patient. Solid lines represent patients alive at the last follow-up, whereas dashed lines represent patients who died prior to it.

In the study group, seven patients received only one cycle of regorafenib, five received two cycles, twelve received three cycles, and five continued therapy beyond three cycles. Twenty-three patients discontinued treatment due to disease progression, while one patient discontinued due to treatment-related toxicity. Four patients died before disease progression could be confirmed. One patient voluntarily withdrew from treatment. The treatment course is illustrated in Figure 1.

**Figure 2:**
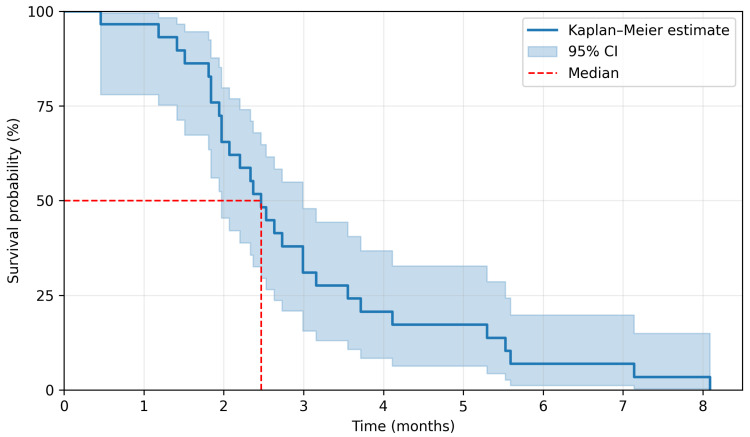
Kaplan–Meier estimation for progression-free survival.

**Figure 3:**
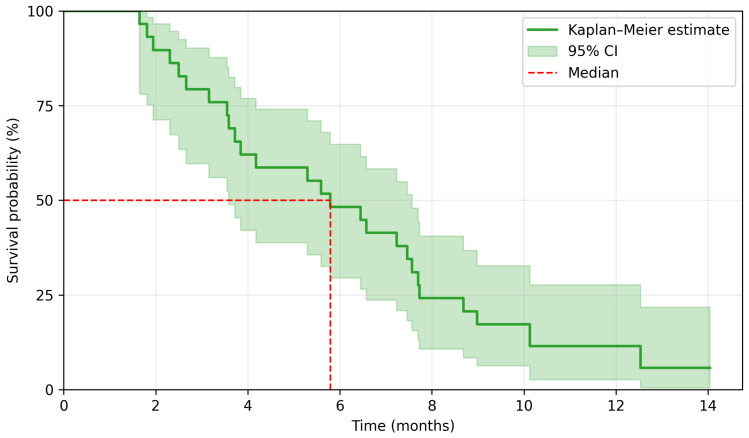
Kaplan–Meier estimation for overall survival.

The median duration of regorafenib treatment was 2.8 months. The last follow-up date for survival was 2 January 2025. There were 29 PFS events and 26 OS events. A median PFS (mPFS) was 2.5 months, and a median OS (mOS) was 5.8 months. The 3-month and 6-month PFS rate was 37.9% and 6.9%, respectively, whereas the 6-month and 12-month OS rate was 48.3% and 6.9%. Kaplan–Meier survival curves are presented in Figure 2 and Figure 3. Among 29 patients, 5 achieved disease stabilization as the best response to therapy, there were no partial or complete remissions. The median duration of disease stabilization was 5.6 months. The comparison of our results with the outcomes of the CORRECT trial is presented in Table 2.

## 4. Discussion

Managing treatment for patients in good performance status who have already received all standard therapies and whose tumors do not exhibit molecular aberrations amenable to targeted therapies is challenging. The CORRECT trial, published in 2013, provided evidence of the efficacy of regorafenib in such patients. In this study, regorafenib was shown to significantly improve OS and PFS compared to placebo. The use of regorafenib reduced the risk of disease progression by 51% (HR 0.49, 95% CI 0.42–0.58; *p* < 0.0001) and the risk of death by 23% (HR 0.77, 95% CI 0.64–0.94; *p* = 0.0052), extending the median PFS from 1.7 to 1.9 months and the median OS from 5 to 6.4 months. No patients achieved a complete response, while 5 patients receiving regorafenib achieved a partial response. Disease control was observed in 41% of patients, with a median duration of disease stabilization of 2 months compared to 1.7 months in the placebo group. A meta-analysis combining results from the CORRECT trial and three studies conducted in Asian populations [12,13,14] demonstrated a statistically significant improvement in OS (OR = 0.78; 95% CI = 0.65–0.94; *p* = 0.008) and PFS (OR = 0.52; 95% CI = 0.34–0.79; *p* = 0.002) compared with the control group [15]. The efficacy and safety of regorafenib have also been supported by European real-world data, including the French compassionate-use REBECCA cohort [16] and the Czech national registry-based analysis [17]. These findings are complemented by the international prospective observational CORRELATE study, which included a substantial number of European centers [18], as well as separate French cohort analysis [19]. Regorafenib was also investigated in the second-line setting in a phase II study that compared FOLFIRI combined with regorafenib versus FOLFIRI alone. The study demonstrated only a slight improvement in PFS (median: 6.1 vs. 5.3 months; HR 0.73, 95% CI = 0.53–1.01; *p* = 0.056); however, no benefit in OS was observed (HR 1.01, 95% CI = 0.71–1.44) [20]. In the Italian REALITY (GISCAD) study [21], clinical factors potentially identifying patients with a greater likelihood of benefit were explored, with the absence of liver progression and good early tolerability (without dose/schedule modifications) associated with improved outcomes. The patient characteristics in these cohorts appear broadly comparable to those in CORRECT and in our analysis. To the best of our knowledge, Polish centers were not represented in the above-mentioned studies.

The outcomes of the study conducted on the population from Polish oncology centers were comparable to those in the CORRECT trial. However, reliable comparison is not possible, as patients treated in real-word practice usually differ from those treated in controlled clinical trials. In the analyzed group, most patients had a performance status of 1 according to ECOG, compared to the registration trial, where patients with good and very good performance status were more evenly distributed. Additionally, the Polish population had a higher prevalence of rectal cancer—more than half compared to one-third of the CORRECT trial population with this diagnosis. Moreover, 34.5% of patients in the current study received more than three lines of treatment prior to regorafenib, compared to 49% in the 2013 trial. Thus, small numerical differences in mPFS and mOS seem to be negligible. Moreover, the difference in median PFS may be attributed to the timing of treatment response assessments, performed every 8 weeks in the registration trial but every 12 weeks in our study. Differences between the size of populations may also contribute to the observed discrepancies. The potential impact of improved supportive care quality for oncology patients since the time of the registration trial should also be considered.

For patients progressing on regorafenib or after exhausting available treatment options, fruquintinib emerges as a promising option. Approved by the FDA in 2023 [22] and by the EMA in June 2024 [23], fruquintinib is a selective VEGFR 1–3 kinase inhibitor that exerts its antitumor effects by inhibiting tumor angiogenesis. Its efficacy was confirmed in the phase III FRESCO-2 trial [24], a randomized study comparing fruquintinib + best supportive care (BSC) with placebo + BSC. This trial included 691 patients with metastatic colorectal cancer previously treated with standard therapies, including fluoropyrimidine, oxaliplatin, irinotecan, anti-EGFR agents (for *KRAS*, *NRAS*, and *BRAF* wild-type tumors), anti-VEGF agents, trifluridine/tipiracil, and immunotherapy for MSI-H tumors. Patients were stratified by prior therapy (trifluridine/tipiracil only, regorafenib only, or both), RAS genes status, and metastatic disease duration (≤18 months or >18 months). The primary endpoint was OS, with PFS as a key secondary endpoint. Fruquintinib significantly improved both endpoints, with a mOS of 7.4 months compared to 4.8 months in the placebo group (34% reduction in death risk) and a mPFS of 3.7 months versus 1.8 months (68% reduction in progression or death risk).

Another treatment approach after standard therapies could involve reintroducing previously used regimens [25]. Evidence suggests that irinotecan-based retreatment achieves a disease control rate (DCR) of 78.2%, compared to 57.8% for oxaliplatin retreatment [26,27]. However, oxaliplatin retreatment may not be feasible due to persistent neuropathy from prior therapy. Reintroduction of anti-EGFR agents in unselected patients is considered ineffective [28].

## 5. Limitations of Our Study

The main limitation of our study is a very small number of patients. This results from the limited availability of regorafenib at that time.

## 6. Conclusions

This analysis focused on patients who had completed standard treatment. The efficacy of regorafenib was evaluated in a population of Polish patients with metastatic colorectal cancer. Despite being a preliminary evaluation based on a small patient cohort our study confirmed the results of the CORRECT trial. Decisions regarding treatment after standard therapies remain challenging and, given the current state of knowledge, should be guided by the anticipated benefits and the side effect profile of available options. Fruquintinib has recently emerged as an additional important therapeutic option. Further comparative studies are necessary to determine the optimal treatment strategies in patients with chemorefractory disease.

## Figures and Tables

**Table 1:** Clinical characteristics of the studied population.

		n	[%]
Patients			
Sex	Male	22	75.9%
	Female	7	24.1%
ECOG	0	9	31.0%
	1	20	69.0%
Weight loss	<10%	10	34.5%
	≥10%	5	17.2%
	No weight loss	14	48.3%
Disease characteristics			
Primary site of disease	Colon	12	41.4%
	Rectum	16	55.2%
	Both	1	3.4%
Grade	Grade 1	9	31.0%
	Grade 2	17	58.6%
	Grade 3	0	0.0%
	Grade unknown	3	10.3%
Metastases	Liver	25	86.2%
	Other *	4	13.8%
Biomarkers			
*KRAS*/*NRAS* mutation	Yes	17	58.6%
	No	11	37.9%
	Unknown	1	3.4%
*BRAF* mutation	Yes	1	3.4%
	No	27	93.2%
	Unknown	1	3.4%
MSI status	MSI-H	0	0.0%
	MSI-L	28	96.6%
	Unknown	1	3.4%
Previous treatment of locally advanced disease			
Resection	Yes	21	72.4%
	No	7	27.6%
Perioperative oxaliplatin-based chemotherapy	Yes	12	41.4%
	No	17	58.6%
Previous treatment of metastatic disease			
Number of previous treatment cycles	≤3	19	65.5%
	>3	10	34.5%
Irinotecan	Yes	29	100.0%
	No	0	0.0%
Oxaliplatin	Yes	29	100.0%
	No	0	0.0%
Trifluridine + Tipiracil	Yes	28	96.6%
	No	1	3.4%
Cetuximab	Yes	9	31.0%
	No	20	69.0%
Panitumumab	Yes	2	6.9%
	No	27	93.1%
Bevacizumab	Yes	14	48.3%
	No	15	51.7%
Aflibercept	Yes	0	0.0%
	No	29	100.0%

* any distant metastatic sites other than the liver.

**Table 2:** Comparison of current results with CORRECT Trial results; mPFS—median progression-free survival, mOS—median overall survival, mDoT—median duration of treatment, mDoSD—median duration of stable disease.

	Current Study	CORRECT
mPFS [months]	2.5	1.9
mOS [months]	5.8	6.4
6-month OS rate [%]	48.3	52.5
12-month OS rate [%]	6.9	24.3
mDoT [months]	2.8	1.7
mDoSD [months]	5.6	2.0

## Data Availability

The data and materials used in this study are available from the corresponding authors upon reasonable request.

## Abbreviations

The following abbreviations are used in this manuscript:

anti-EGFR	anti-Epidermal Growth Factor Receptor
anti-VEGF	anti-Vascular Endothelial Growth Factor
BSC	Best Supportive Care
DCR	Disease Control Rate
ECOG	Eastern Cooperative Oncology Group
EMA	European Medicines Agency
ESMO	European Society for Medical Oncology
FDA	Food and Drug Administration
HER2	Human Epidermal Growth Factor Receptor 2
HR	Hazard Ratio
mDoSD	median Duration of Stable Disease
mDoT	median Duration of Treatment
mOS	median Overall Survival
mPFS	median Progression-Free Survival
MSI	Microsatellite Instability
MSI-H	Microsatellite Instability-High
MSI-L	Microsatellite Instability-Low
OS	Overall Survival
PFS	Progression-Free Survival
RAS	Rat sarcoma
VEGFR	Vascular Endothelial Growth Factor Receptor
